# Atypical Presentation of Drug Reaction With Eosinophilia and Systemic Symptoms (DRESS) Syndrome: When Gastrointestinal Symptoms Obscure the Diagnosis

**DOI:** 10.7759/cureus.69581

**Published:** 2024-09-17

**Authors:** Khine Min Thaw, Eingyin Ko Ko, Ashar U Kazi

**Affiliations:** 1 Acute Medicine, Pilgrim Hospital Boston, Boston, GBR

**Keywords:** delayed hypersensitivity, drug-induced hypersensitivity, drug reaction with eosinophilia and systemic symptoms (dress) syndrome, exanthematous drug eruption, regiscar scoring system, stevens-johnson syndrome (sjs)

## Abstract

The drug reaction with eosinophilia and systemic symptoms (DRESS) syndrome is a severe cutaneous adverse reaction. Due to its unfamiliarity, non-specific diagnostic criteria, and delayed onset, this condition is frequently overlooked, which can sometimes result in life-threatening consequences.

DRESS typically manifests as an extensive mucocutaneous rash with multi-organ involvement. This report aims to emphasize the varied presentation of the syndrome. Our patient was initially presented with acute onset vomiting, abdominal pain, fever for a couple of days with a minor skin rash. At first, she was treated for acute viral gastritis. However, on her second presentation within a week, she had a more extensive skin rash. Upon detailed history, it was found that this was linked to the initiation of sulfasalazine for ulcerative colitis. RegiSCAR (Registry of Severe Cutaneous Adverse Reactions) scoring suggested it as a definite case of DRESS.

The primary manifestation of gastrointestinal symptoms led to a delayed diagnosis. Still, it is important to consider the possibility of drug hypersensitivity when there are skin changes and blood abnormalities present.

## Introduction

Drug-induced hypersensitivity syndrome (DIHS), or drug reaction with eosinophilia and systemic symptoms (DRESS) syndrome, is a severe condition with an estimated prevalence of 2.18 per 100,000 patients and a mortality rate of 10% [1,2]. The exact cause is unclear, with proposed mechanisms involving detoxification enzyme deficiency, delayed immune response, and genetic factors [2]. Sulfasalazine is the most common offending drug, while antibiotics are the drug class most frequently involved. The symptoms typically manifest between two to six weeks, with a median onset time of 24 days. It is one of the severe cutaneous adverse reactions (SCAR) and presents with a variety of symptoms, including widespread rashes, fever, and multi-organ involvement, particularly affecting the liver [3].

Apart from the liver, the gastrointestinal tract (GIT) is seldom involved in DRESS syndrome. The pancreas was the most commonly affected GI organ, often leading to type 1 diabetes or chronic pancreatic insufficiency. Meanwhile, the stomach is involved in only 1.9% of cases but vomiting and diarrhea are the most frequent GIT symptoms, occurring in 19.6% of cases each [4]. Conversely, life-threatening manifestations such as abrupt GI bleeding can also develop as a part of DRESS syndrome [5].

Diagnosis is challenging and is based on clinical judgment, aided by the RegiSCAR (Registry of Severe Cutaneous Adverse Reactions) scoring system [6]. Corticosteroids remain the mainstay of treatment, while immunosuppressive therapies are reserved for severe cases [3,7-9].

## Case presentation

An Asian lady in her mid-20s presented with vomiting, abdominal pain, and a fever of 38.6°C for two days. Clinical assessment showed signs of dehydration and mild tenderness in the upper abdomen. Blood tests revealed a slightly elevated C-reactive protein (CRP) level of 30.79 and a platelet count of 130. During her hospital stay, a sparsely distributed, small, red, papular skin rash resembling viral exanthema developed on her trunk, limbs, and palms. After receiving an intravenous (IV) proton pump inhibitor, fluids, and antiemetics for acute viral gastritis, the patient was discharged from the hospital 48 hours later.

**Figure 1:**
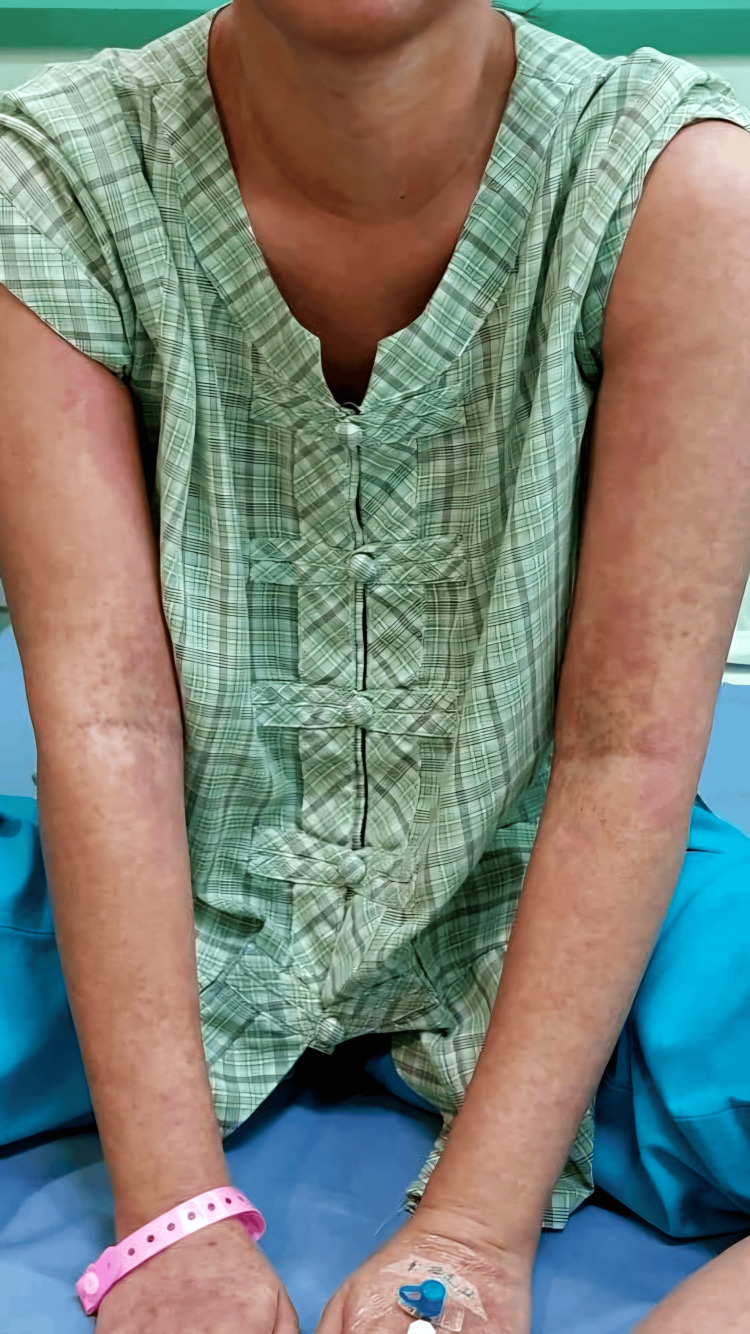
Maculopapular rash on the neck and arms during the patient’s second presentation to the emergency department.

**Figure 2:**
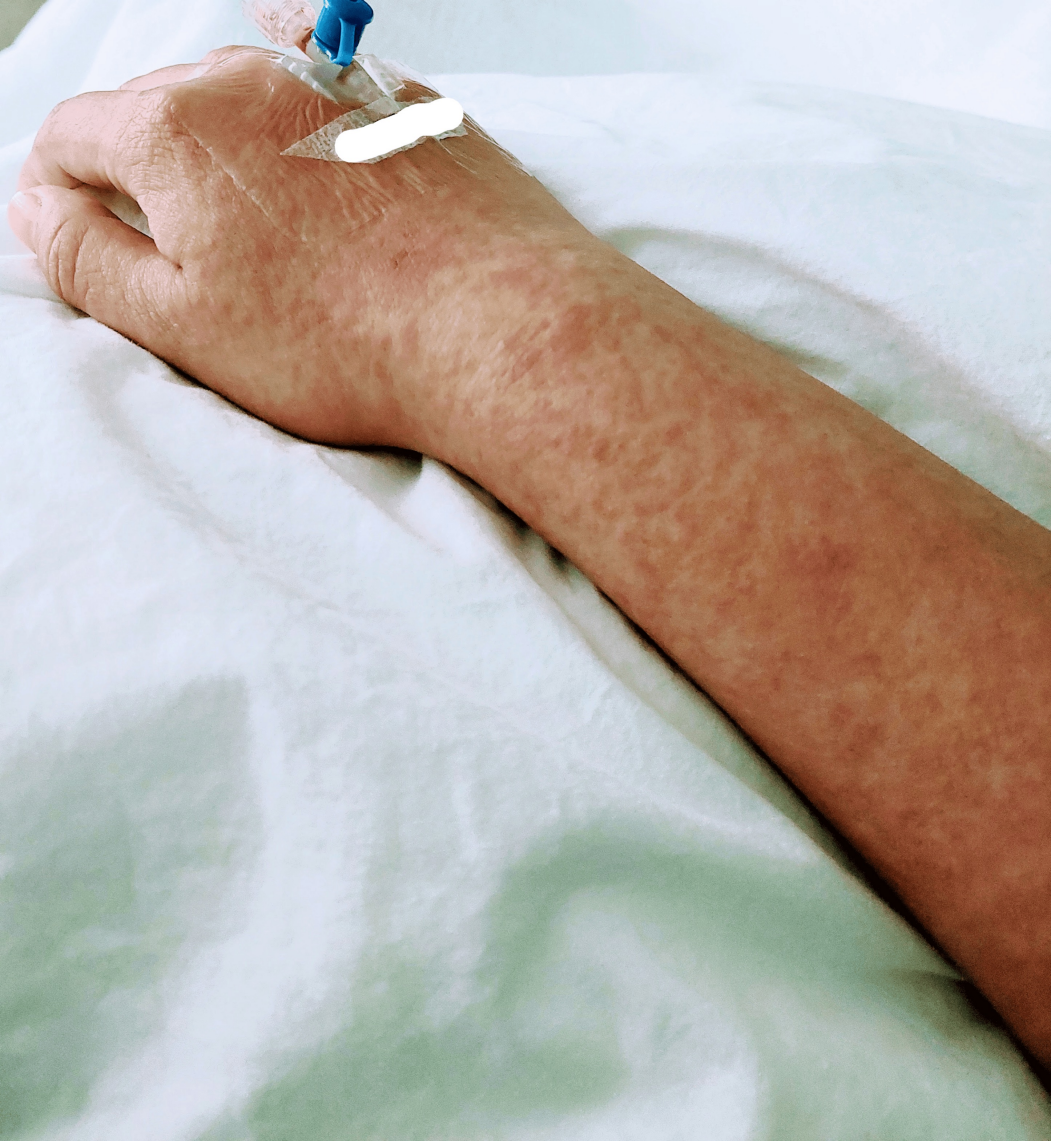
Maculopapular rash on the right forearm during the patient’s second presentation to the emergency department.

However, five days post-discharge, the patient revisited the emergency department presenting identical symptoms alongside a more pronounced skin rash with severe itching (Figures 1-2). Clinical evaluation revealed that the red maculopapular rash had extended to over half of her body surface area, encompassing her face and neck. Additional observations included jaundiced sclera, palpable lymph nodes in her neck: swollen beyond 1 cm, purpura on both earlobes, and a slightly enlarged liver. No involvement of the mucous membranes was noted.

Investigation

Blood tests revealed a significant increase in eosinophil count (1.15 x 10^3µ/L), abnormal liver function tests, and elevated CRP (Table 1: Second presentation). Upon further discussion, the patient reported no known drug allergies. However, she started sulfasalazine five weeks prior for newly diagnosed ulcerative colitis and was not on any other regular medications. The antinuclear antibody (ANA) test returned positive at a 1/400 dilution, with anti-Ro and anti-RNA polymerase antibodies in the extractable nuclear antigen (ENA) profile.

**Table 1:** Blood test results

Blood tests	Normal range	Unit	First presentation (day 2)	Second presentation (day 9)	Third day of systemic steroid (day 12)
C-reactive protein (CRP)	0-5	mg/L	30.79	63.9	9.35
White cell count	4.3-11.2	10^3µ/L	7.10	15.8	16.16
Neutrophil count	2.1-7.4	10^3µ/L	4.08	7.25	6.41
Eosinophil count	0.02-0.5	10^3µ/L	0.27	1.15	0.03
Total bilirubin	0-21	µmol/L	-	29.22	1.032
Aspartate aminotransferase (AST)	8-33	U/L	-	98.49	35.6
Alanine aminotransferase (ALT)	0-33	U/L	-	189.3	105.6
Alkaline phosphatase (ALP)	30-130	U/L	-	214	168

Differentials

As the patient was presented initially with fever and a skin rash thought to be viral exanthema, she was tested for Dengue antigen and antibodies. The test showed positive IgG only, indicating that the patient did not have an active dengue infection. Further viral screens for hepatitis B and C, and HIV all came back negative. While Epstein-Barr virus (EBV) and cytomegalovirus (CMV) infections were suspected, these were not ultimately tested.

The appearance of a widespread itchy skin rash accompanied by the involvement of several organs such as the liver, GIT, and lymph nodes suggested a cutaneous drug reaction. Initially, Stevens-Johnson syndrome (SJS) was considered as a likely cause but the eosinophilic pattern in the full blood count pointed towards DRESS syndrome. Moreover, sulfasalazine is one of the most notorious medications that frequently causes this syndrome.

The RegiSCAR score [6] was calculated and was shown to be 6 (definite case) due to the presence of fever, lymphadenopathy, extensive skin rash, eosinophilia, facial oedema, purpura, and involvement of GIT and liver. Therefore, the final diagnosis of sulfasalazine-induced DRESS syndrome was made.

Treatment

The patient received systemic steroid therapy, starting with IV hydrocortisone at 100 mg immediately followed by four times daily for 24 hours. This was subsequently changed to IV methylprednisolone pulse therapy: 500 mg once daily for three days, followed by 125 mg once daily for another three days. Sulfasalazine was stopped at once, and the gastroenterologist switched it to mesalazine after three to four weeks of steroid therapy. The dermatologist reviewed the case and agreed with the diagnosis of DRESS syndrome and the ongoing treatment plan. With a positive ENA profile, the rheumatologist identified it as an undifferentiated connective tissue disorder that is not threatening to organs. No additional treatment was prescribed since the patient is already receiving mesalazine, which is also a disease-modifying antirheumatic drug (DMARD), for ulcerative colitis.

Follow-up

The patient showed gradual recovery with the treatment described above, as evidenced by a good response in blood results (Table 1: Third day of systemic steroid), and was discharged after seven days with oral prednisolone tapering dose over eight weeks. Consistent improvement in symptoms and blood results was observed during follow-up appointments at the first, third, and eight-week post-discharge. The skin rash gradually diminished and almost completely cleared by day 20 from symptom onset. Thyroid function tests (thyroid stimulating hormone, free T3 and T4) were checked at the eight-week follow-up to monitor for autoimmune thyroiditis, and the results were completely normal.

## Discussion

DRESS syndrome is a type of SCAR, that needs to be distinguished from acute generalized exanthematous pustulosis (AGEP), SJS, and toxic epidermal necrolysis (TEN) due to their immediate and life-threatening nature. However, DRESS is not as commonly identified in everyday clinical practice. AGEP usually manifests within 24-48 hours after starting the offending medication [10]. SJS and TEN are more probable if the reaction starts within 5 to 28 days of drug administration [11]. In contrast, the onset of DRESS can vary, typically averaging two to six weeks, although there are reports of cases beginning after eight weeks [3]. In our case, symptoms appeared after five weeks of medication use, which lowered our suspicion of SJS/TEN. Nevertheless, DRESS syndrome was initially overlooked due to its rarity and subtler presentation.

Clinicians would likely have been more vigilant if the initial symptom had been a skin rash, as 99% of DRESS syndrome cases present with this feature. However, DRESS syndrome can appear in different ways. In the case discussed, incessant vomiting was the primary symptom that prompted the patient to seek emergency care, rather than a rash. While 8% of cases may exhibit GI symptoms [3], the most likely differential diagnosis in a young adult would typically be acute gastroenteritis. Nevertheless, the emergence of skin rashes should prompt further investigation. Viral exanthema, potentially linked to various viruses including Kawasaki disease, EBV, hepatitis viruses, influenza virus, CMV, and HIV, must also be considered. Moreover, when a patient presents with a pruritic maculopapular rash accompanied by high fever, a thorough medication history is crucial, and drug-induced skin reactions must be considered among the differential diagnoses. In fact, it is crucial to identify and scrutinize all medications that were initiated within the preceding eight weeks of symptom onset [12]. Therefore, it is advised to maintain a high level of clinical suspicion to achieve a prompt diagnosis and appropriate treatment.

At present, the diagnosis of DRESS syndrome mainly relies on the clinical judgment correlating signs and laboratory findings. Currently, there is no gold standard for diagnostic criteria or single definitive laboratory test to diagnose the syndrome. The RegiSCAR scoring system for DRESS is one of the primary tools used to assist in clinical diagnosis. Furthermore, different study groups have proposed different scoring systems to classify the severity of the syndrome [7,13,14]. These scoring systems can assist clinical judgment. The skin patch test is one of the recommended tests to find out the culprit medication [7], but it should be aware that different medications may have different effects on the result. In our case, sulfasalazine would not yield a positive skin patch result [15]. The intradermal test is recommended if the skin patch test is negative, but the availability of IV formulation may influence the use of this test [16]. Overall, the basic workup for the syndrome should include full blood count, liver function tests, renal function tests, urine sediments, and ECG [9]. The tests can be extended to include coagulation profile, ANA, ENA, organ biopsy (skin/liver), pulmonary function tests, pancreatic enzymes, and 24-hour urinary protein to detect organ involvement early [8,9].

To our knowledge, there are no internationally agreed management guidelines to this date. The 2024 Delphi-based consensus would be one of the first steps towards establishing one. Systematic reviews largely endorse corticosteroids as the initial therapy, in conjunction with stopping the offending medication. The RegiSCAR score may guide the choice of treatment on the type and administration route of corticosteroids, based on the severity of internal organ involvement or a RegiSCAR score of < 4 or ≥ 5 [2]. Depending on the severity, the French guideline advised using topical or systemic steroids, with or without intravenous immunoglobulins (IVIGs) or antivirals [8]. The Spanish guideline made a stepwise approach to treatment, mainly based on the severity of drug-induced liver injury (DILI) [7]. The Delphi-based consensus agrees to start IV corticosteroids with more than one organ involvement in addition to the liver or kidney [9]. Pulse methylprednisolone therapy, as in our case, may be used to control acute phase symptoms and also to prevent autoimmune disease from developing later on [17,18]. This rationale has been disputed in a recent study which found that steroid pulse therapy can be associated more with CMV reactivation, symptom persistence, and high mortality, compared to conventional oral steroids [19]. However, our patient recovered without complications.

Lastly, close monitoring of patients for potential DRESS relapses and its long-term consequences, including autoimmune disorders such as thyroiditis or type 1 diabetes, and end-organ failure [5], is crucial, especially in the elderly. The Delphi-based consensus agrees that patients should be followed up closely within the first month, followed by regular checks within the first six months after onset to screen for autoantibodies and thyroid dysfunction, and to monitor steroid side effects [9].

## Conclusions

DRESS syndrome can manifest in different forms. The presence of a skin rash and fever should prompt healthcare providers to suspect a drug-induced skin reaction and watch for possible internal organ involvement. When DRESS syndrome is suspected, tools like the RegiSCAR score or similar systems can help make a diagnosis and support research. These steps will ultimately aid future healthcare teams in creating effective management protocols.

## References

[1] Wolfson AR, Zhou L, Li Y, Phadke NA, Chow OA, Blumenthal KG (2019). Drug reaction with eosinophilia and systemic symptoms (DRESS) syndrome identified in the electronic health record allergy module. J Allergy Clin Immunol Pract.

[2] De A, Rajagopalan M, Sarda A, Das S, Biswas P (2018). Drug reaction with eosinophilia and systemic symptoms: an update and review of recent literature. Indian J Dermatol.

[3] Awad A, Goh MS, Trubiano JA (2023). Drug reaction with eosinophilia and systemic symptoms: a systematic review. J Allergy Clin Immunol Pract.

[4] Jevtic D, Dumic I, Nordin T (2021). Less known gastrointestinal manifestations of drug reaction with eosinophilia and systemic symptoms (DRESS) syndrome: a systematic review of the literature. J Clin Med.

[5] Kano Y, Ishida T, Hirahara K, Shiohara T (2010). Visceral involvements and long-term sequelae in drug-induced hypersensitivity syndrome. Med Clin North Am.

[6] Kardaun SH, Sidoroff A, Valeyrie-Allanore L, Halevy S, Davidovici BB, Mockenhaupt M, Roujeau JC (2007). Variability in the clinical pattern of cutaneous side-effects of drugs with systemic symptoms: does a DRESS syndrome really exist?. Br J Dermatol.

[7] Cabañas R, Ramírez E, Sendagorta E (2020). Spanish guidelines for diagnosis, management, treatment, and prevention of DRESS syndrome. J Investig Allergol Clin Immunol.

[8] Descamps V, Ben Saïd B, Sassolas B (2010). Management of drug reaction with eosinophilia and systemic symptoms (DRESS) [Article in French]. Ann Dermatol Venereol.

[9] Brüggen MC, Walsh S, Ameri MM (2024). Management of adult patients with drug reaction with eosinophilia and systemic symptoms: a Delphi-based international consensus. JAMA Dermatol.

[10] Parisi R, Shah H, Navarini AA, Muehleisen B, Ziv M, Shear NH, Dodiuk-Gad RP (2023). Acute generalized exanthematous pustulosis: clinical features, differential diagnosis, and management. Am J Clin Dermatol.

[11] Oakley AM, Krishnamurthy K (2023). Stevens-Johnson syndrome. StatPearls [Internet].

[12] Hama N, Abe R, Gibson A, Phillips EJ (2022). Drug-induced hypersensitivity syndrome (DIHS)/drug reaction with eosinophilia and systemic symptoms (DRESS): clinical features and pathogenesis. J Allergy Clin Immunol Pract.

[13] Momen SE, Diaz-Cano S, Walsh S, Creamer D (2021). Discriminating minor and major forms of drug reaction with eosinophilia and systemic symptoms: facial edema aligns to the severe phenotype. J Am Acad Dermatol.

[14] Shiohara T, Iijima M, Ikezawa Z, Hashimoto K (2007). The diagnosis of a DRESS syndrome has been sufficiently established on the basis of typical clinical features and viral reactivations. Br J Dermatol.

[15] Barbaud A, Collet E, Milpied B (2013). A multicentre study to determine the value and safety of drug patch tests for the three main classes of severe cutaneous adverse drug reactions. Br J Dermatol.

[16] Teo YX, Friedmann PS, Polak ME, Ardern-Jones MR (2023). Utility and safety of skin tests in drug reaction with eosinophilia and systemic symptoms (DRESS): a systematic review. J Allergy Clin Immunol Pract.

[17] Chiou CC, Yang LC, Hung SI (2008). Clinicopathological features and prognosis of drug rash with eosinophilia and systemic symptoms: a study of 30 cases in Taiwan. J Eur Acad Dermatol Venereol.

[18] Husain Z, Reddy BY, Schwartz RA (2013). DRESS syndrome: part II. Management and therapeutics. J Am Acad Dermatol.

[19] Hashizume H, Ishikawa Y, Ajima S (2022). Is steroid pulse therapy a suitable treatment for drug-induced hypersensitivity syndrome/drug reaction with eosinophilia and systemic symptoms? A systematic review of case reports in patients treated with corticosteroids in Japan. J Dermatol.

